# Relative contributions of sex hormones, sex chromosomes, and gonads to sex differences in tissue gene regulation

**DOI:** 10.1101/gr.275965.121

**Published:** 2022-05

**Authors:** Montgomery Blencowe, Xuqi Chen, Yutian Zhao, Yuichiro Itoh, Caden N. McQuillen, Yanjie Han, Benjamin L. Shou, Rebecca McClusky, Karen Reue, Arthur P. Arnold, Xia Yang

**Affiliations:** 1 Department of Integrative Biology and Physiology, University of California, Los Angeles, California 90095, USA;; 2 Interdepartmental Program of Molecular, Cellular and Integrative Physiology, University of California, Los Angeles, California 90095, USA;; 3 Laboratory of Neuroendocrinology of the Brain Research Institute, University of California, Los Angeles, California 90095, USA;; 4 Department of Neurology, David Geffen School of Medicine, University of California, Los Angeles, California 90095, USA;; 5 Department of Human Genetics and Molecular Biology Institute, David Geffen School of Medicine, University of California, Los Angeles, California 90095, USA;; 6 Institute for Quantitative and Computational Biosciences, University of California, Los Angeles, California 90095, USA;; 7 Molecular Biology Institute, University of California, Los Angeles, California 90095, USA

## Abstract

Sex differences in physiology and disease in mammals result from the effects of three classes of factors that are inherently unequal in males and females: reversible (activational) effects of gonadal hormones, permanent (organizational) effects of gonadal hormones, and cell-autonomous effects of sex chromosomes, as well as genes driven by these classes of factors. Often, these factors act together to cause sex differences in specific phenotypes, but the relative contribution of each and the interactions among them remain unclear. Here, we used the four core genotypes (FCG) mouse model with or without hormone replacement to distinguish the effects of each class of sex-biasing factors on transcriptome regulation in liver and adipose tissues. We found that the activational hormone levels have the strongest influence on gene expression, followed by the organizational gonadal sex effect, and last, sex chromosomal effect, along with interactions among the three factors. Tissue specificity was prominent, with a major impact of estradiol on adipose tissue gene regulation and of testosterone on the liver transcriptome. The networks affected by the three sex-biasing factors include development, immunity and metabolism, and tissue-specific regulators were identified for these networks. Furthermore, the genes affected by individual sex-biasing factors and interactions among factors are associated with human disease traits such as coronary artery disease, diabetes, and inflammatory bowel disease. Our study offers a tissue-specific account of the individual and interactive contributions of major sex-biasing factors to gene regulation that have broad impact on systemic metabolic, endocrine, and immune functions.

Females and males differ in the risk, incidence, and progression of complex diseases such as obesity, nonalcoholic fatty liver disease, and diabetes (Arnold 2010; Clayton and Collins 2014; Rask-Andersen et al. 2019). Thus, one sex may have endogenous protective or risk factors that could become targets for therapeutic interventions. Current sexual differentiation theory suggests that three major classes of factors cause sex differences (Arnold 2009, 2012, 2017; Arnold et al. 2013; Schaafsma and Pfaff 2014). First, some sex differences are caused by different circulating levels of ovarian and testicular hormones, known as “activational effects.” These differences are reversible because they are eliminated by gonadectomy of adults. Second, certain sex differences persist after gonadectomy in adulthood and represent the effects of permanent or differentiating effects of gonadal hormones, known as “organizational effects,” that form during development. A third class of sex differences are caused by the inequality of action of genes on the X and Y Chromosomes in male (XY) and female (XX) cells, and these are called “sex chromosome effects.”

To date, few studies have systematically evaluated the relative importance of these three classes of factors acting on phenotypic or gene regulation systems (Arnold 2019). The activational effects of hormones have been established as a significant contributor to sexual dimorphism in metabolic diseases, with additional evidence pointing to sex chromosome effects on obesity and lipid metabolism (Chen et al. 2012; Link et al. 2015, 2020). Previous studies have also emphasized the importance of organizational or activational hormone effects on liver gene expression (Mode and Gustafsson 2006; van Nas et al. 2009; Waxman and Holloway 2009; Sugathan and Waxman 2013; Zheng et al. 2018). However, the tissue-specific contributions and the interactions of activational, organizational, and sex chromosome effects on gene regulation are poorly investigated.

Here, we conduct a systematic investigation to understand the relative contribution of the three sex-biasing factors in gene regulation (Fig. 1). We used the four core genotypes (FCG) mouse model, in which the type of gonad (ovary or testis) is independent of sex chromosome complement (XX or XY) (De Vries et al. 2002; Burgoyne and Arnold 2016). The model separates the effects of sex chromosome complement by fixing the gonadal status (XX vs. XY with ovaries; XX vs. XY with testes) from the effects of gonads by fixing the sex chromosome type (ovaries vs. testes with XX genotype; ovaries vs. testes with XY genotype). By varying adult gonadal hormone levels via gonadectomy and subsequent hormonal treatments, we also asked how androgens and estrogens influence gene expression as a function of sex chromosome complement and gonadal sex. The design allows comparison of the magnitude of effect of each sex-biasing factor and the interactions among different factors.

**Figure 1.**
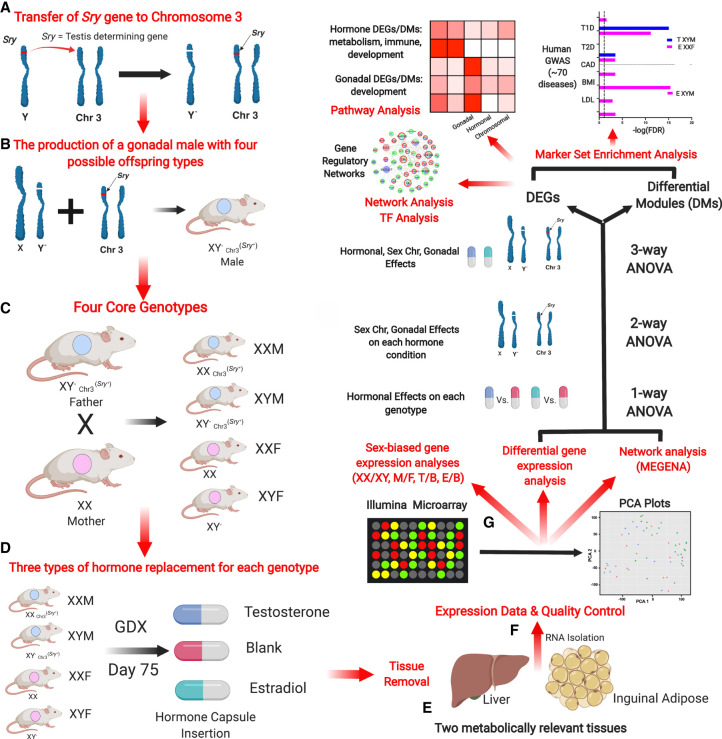
Overall study design. (*A*) Transfer of the *Sry* gene to Chromosome 3. *Sry*, which is usually located on the Y Chromosome, was deleted (a spontaneous deletion) and inserted as a transgene onto Chromosome 3, making *Sry* independent of the Y Chromosome. (*B*) The production of a gonadal male XY^−^_Chr3_^*Sry*+^, which has the ability to produce four types of gametes resulting in the four core genotypes (FCG). (*C*) The generation of the FCG mice. Mating of XY^−^_Chr3_^*Sry*+^ male and XX female produces four types of mouse offspring (two gonadal males and two gonadal females): XY^−^_Chr3_^*Sry*+^ (XYM), XX_Chr3_^*Sry*+^ (XXM), XX (XXF), XY^−^ (XYF). (*D*) Modulation of sex hormones in mouse offspring of each genotype after gonadectomy (GDX). Each of the four core genotypes underwent GDX at day 75 and was implanted with a capsule that contained either estradiol or testosterone, or was blank (n = 5/genotype/treatment). (*E*) Dissection of the liver and inguinal adipose tissue for RNA isolation. (*F*) Gene expression profiling and quality control. Using an Illumina microarray, we measured the transcriptome and then performed a principal component analysis (PCA) to identify outliers and global patterns. (*G*) Bioinformatics analyses. Differentially expressed genes (DEGs) influenced by individual sex-biasing factors were identified using three-way ANOVA (chromosomal, gonadal, and hormonal effects), two-way ANOVA (gonadal and chromosomal effects under each hormone condition), and a one-way ANOVA (estradiol and testosterone treatment effects in individual genotypes). Gene coexpression networks were constructed using MEGENA and differential coexpression modules (DMs) affected by individual sex-biasing factors were identified using three-way, two-way, and one-way ANOVAs. DEGs and DMs were analyzed for enrichment of functional categories or biological pathways. The relevance of the DEGs to human disease was assessed via integration with human genome-wide association studies (GWAS) for more than 70 diseases using the marker set enrichment analysis (MSEA). Transcription factor analysis and gene regulatory network analysis were additionally conducted on the DEGs derived from the one-way ANOVA.

Using the FCG model, our aim is to assess the role of the three sex-biasing factors and their interactions on gene expression, molecular pathways, and gene network organization in the liver and adipose tissue, which are central tissues for metabolic and endocrine homeostasis, with adipose tissue additionally contributing to immune functions. We further aim to understand the relationship of each sex-biasing factor with various human diseases.

## Results

### Overall study design

In FCG mice, the Y Chromosome (from strain 129) has sustained a spontaneous deletion of *Sry*, and an *Sry* transgene is inserted onto Chromosome 3 (Fig. 1A) to produce gonadal male XY^−^_Chr3_^*Sry*^^+^ (Fig. 1B; Burgoyne and Arnold 2016). Here, “male” (M) refers to a mouse with testes, and “female” (F) refers to a mouse with ovaries. FCG mice include XX males (XXM) and females (XXF), and XY males (XYM) and females (XYF) (Fig. 1C). A total of 60 FCG mice were gonadectomized (GDX) at 75 d of age and implanted immediately with medical-grade Silastic capsules containing Silastic adhesive only (blank control; B) or testosterone (T) or estradiol (E) (Fig. 1D). This study design produced 12 groups, with four groups of FCG mice (XXM, XXF, XYM, XYF) and each group subdivided into B, T, or E based on hormonal treatment: XXM_B, XXM_T, XXM_E, XYM_B, XYM_T, XYM_E, XXF_B, XXF_T, XXF_E, XYF_B, XYF_T, XYF_E (n = 5/genotype/treatment). Liver and inguinal adipose tissues were collected 3 wk later for transcriptome analysis (Fig. 1E). All liver samples passed quality control (n = 5/group), and five adipose samples across four of the 12 groups failed quality control (n = 3–5/group) (Methods; Fig. 1F). The design allowed detection of differences caused by three factors contributing to sex differences in traits (Fig. 1G). (1) “Sex chromosome effects” were evaluated by comparing XX and XY groups (n = ∼30/sex chromosome type/tissue). (2) “Gonadal sex effects” were determined by comparing mice born with ovaries versus testes (n = ∼30/gonad type/tissue). Because mice were analyzed as adults after removal of gonads, the gonadal sex effects represent organizational (long-lasting) effects of gonadal hormones, such as those occurring prenatally, postnatally, or during puberty. This group also includes effects of the *Sry* gene, which is present in all mice with testes and absent in those with ovaries. Any direct effects of *Sry* on non-gonadal target tissues would be grouped with effects of gonadal sex. (3) “Hormone treatment effects” refer to the effects of circulating gonadal hormones (activational effects) and were evaluated by comparing E versus B groups for estradiol effects, and T versus B groups for testosterone effects, with n = ∼20/hormone type/tissue.

### Global effects of sex chromosome complement, gonadal sex, and hormonal treatments on liver and adipose tissue gene expression

To visualize the overall gene expression trends caused by effects of the three primary sex-biasing components, we conducted principal component analysis (PCA) (Supplemental Fig. S1). For adipose tissue, hormonal treatment (Supplemental Fig. S1A), sex chromosomes (Supplemental Fig. S1B), and gonadal sex (Supplemental Fig. S1C) did not clearly separate the groups. However, in the liver there was a separation of groups based on gonadal hormones, particularly in response to testosterone treatment (Supplemental Fig. S1D) but not based on chromosomal or gonadal factors (Supplemental Fig. S1E,F).

**Figure 2.**
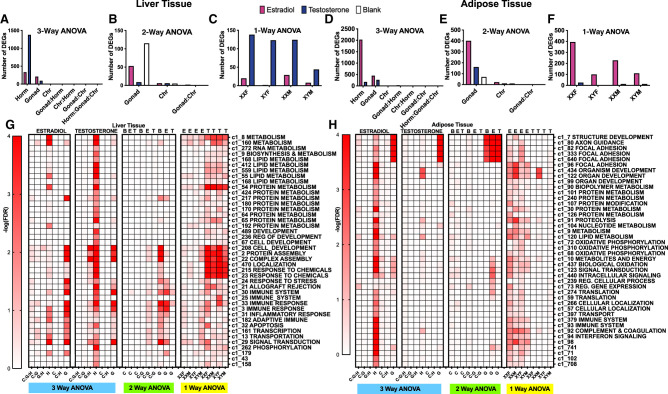
Bar graphs (*A*–*F*) and heatmaps (*G*,*H*) representing the number of DEGs for each sex-biasing factor and differential coexpression modules from a three-way, two-way, and one-way ANOVA, respectively. Each bar graph represents the number of DEGs based on each specific statistical analysis at FDR < 0.05, in liver (*A*–*C*) and inguinal adipose tissue (*D*–*F*). (*A*,*D*) Results from three-way ANOVAs run separately in testosterone versus blank groups, and estradiol versus blank groups to examine hormone, gonad, and sex chromosome effects as well as the interaction terms. Pink bars indicate estradiol versus blank; blue bars indicate testosterone versus blank. (*B*,*E*) Results from two-way ANOVAs with factors of gonadal sex and sex chromosomes as well as the interaction term, run separately on data from testosterone (T), estradiol (E), and blank (B) treatment groups. Colors represent the hormonal treatment condition (testosterone groups blue, estradiol groups pink, and blank groups white). (*C*,*F*) Results from one-way ANOVA testing effects of hormonal treatments (vs. blank) in each of the four genotypes for liver and inguinal adipose tissue. Colors show effects of testosterone versus blank (blue) or estradiol versus blank (pink) in each of the four genotypes. (Horm) hormone, (Chr) sex chromosome, (M) testes/*Sry* present, (F) ovaries present, no *Sry*. (*G*) Coexpression module heatmap for liver. (*H*) Coexpression module heatmap for adipose tissue. Each heatmap shows results from one-way, two-way, and three-way ANOVAs for hormone (H), chromosome (C), and gonad (G) when treated with testosterone (T), estradiol (E), and blank (B). Interaction terms among H, C, and G were also tested. For instance, C:G:H indicates the interaction term among the three factors in three-way ANOVA. The influence of each sex-biasing factor on the coexpression modules was assessed using the first principal component of each module to represent the expression of that module, followed by three-way, two-way, one-way ANOVAs to identify differential modules (DMs) at FDR < 0.05 that are influenced by the various sex-biasing factors. Each module was annotated with canonical pathways from GO and KEGG. Modules without pathway annotations did not show significant enrichment for genes in any pathways tested. Colors correspond to the statistical significance of the effects of sex factors on modules in the form of −log_10_(FDR).

We then asked which individual genes in liver and adipose tissues were affected by adult hormone level, gonadal sex, and sex chromosome complement, as well as interactions between these factors, using three sets of ANOVA tests to address biological questions at different resolution. We defined a differentially expressed gene (DEG) as a gene that passed a false discovery rate (FDR) < 0.05 for individual sex-biasing factors or the interaction terms from the ANOVAs. First, we used a three-way ANOVA (3WA) to test the main effects of sex hormones, gonad type, and sex chromosome as well as the interaction terms. Tens to thousands of DEGs were identified in liver (Table 1) and adipose tissue (Table 2). In both tissues, hormonal treatments affected the largest numbers of genes, followed by fewer genes that were responsive to gonadal/organizational effects or sex chromosome complement (Fig. 2). Testosterone treatment in the liver induced the largest number of DEGs (Fig. 2A), whereas in adipose tissue, estradiol treatment affected the greatest number of DEGs (Fig. 2D). These trends remained when different statistical cutoffs (unadjusted *P* < 0.05, *P* < 0.01, FDR < 0.1, FDR < 0.05) were used (Supplemental Fig. S2). These results support tissue-specific sensitivity to different hormones.

**Table 1.**
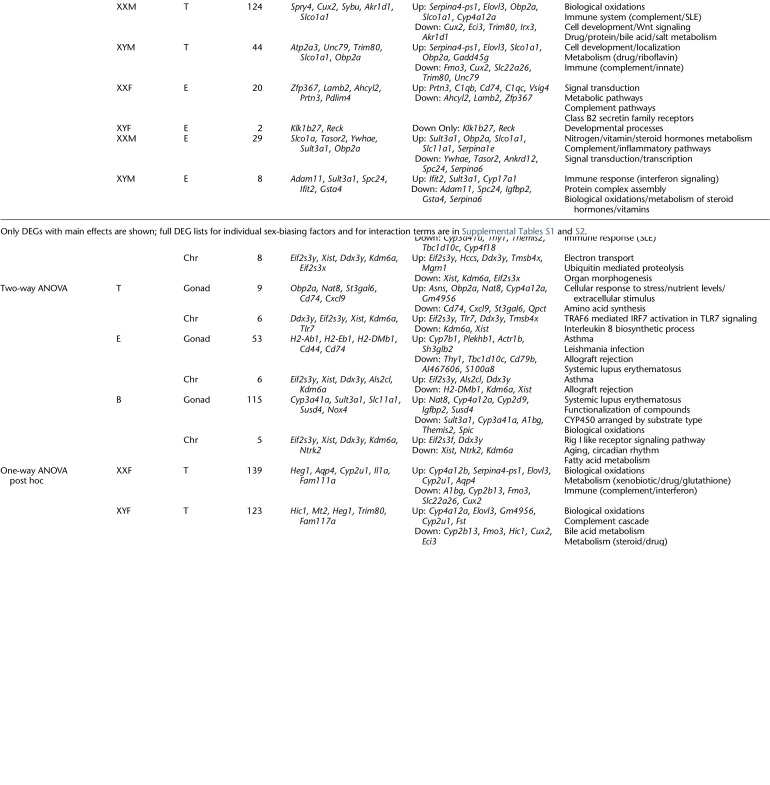
Liver DEGs affected by sex-biasing factors and the associated GO/KEGG pathways

**Table 2.**
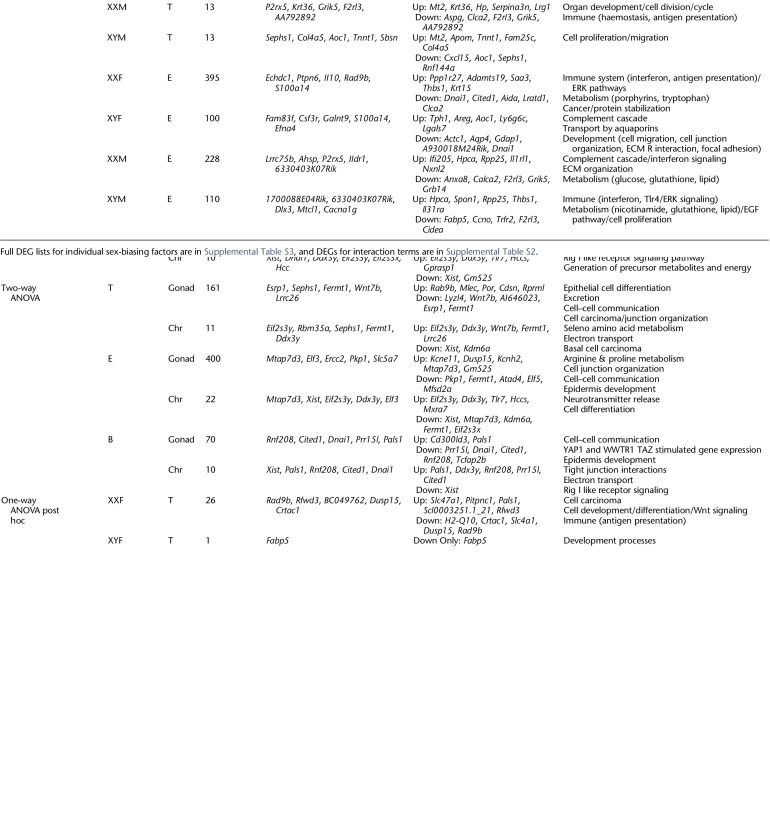
Inguinal adipose DEGs affected by sex-biasing factors and the associated GO/KEGG pathways

Next, we asked if the sex chromosome and gonadal effects are more evident in specific hormonal treatment groups using a two-way ANOVA (2WA). In the liver, the organizational effects of gonad type were strongest in gonadectomized mice without hormone replacement (blank group) (Fig. 2B). In contrast, in adipose tissue the gonadal sex effect was most prominent in the estradiol-treated groups (Fig. 2E), suggesting that estradiol levels augment the enduring differential effects of gonads on the adipose transcriptome. Sex chromosome effects were limited regardless of hormonal treatment status.

Last, we examined whether the effects of testosterone and estradiol are dependent on genotypes using a one-way ANOVA (1WA) followed by post hoc analysis. More liver genes were affected by testosterone than by estradiol regardless of genotype, although XYM liver appeared to be less responsive to testosterone than liver from other genotypes (Fig. 2C). In contrast, in adipose tissue, estradiol affected more DEGs in XX genotypes (XXM and XXF) than in XY genotypes (XYM and XYF), whereas testosterone had minimal impact on adipose tissue gene expression in all four genotypes (Fig. 2F). These results further support tissue-specific effects of estradiol in adipose tissue and testosterone in liver, and indicate that activational effects of hormones also depend on sex chromosome complement and hormonal history (gonadal sex) of the animal.

### Genes and pathways affected by hormonal treatment

In the liver, the 3WA analysis showed that testosterone treatment induced the greatest number of DEGs with 1378 compared to 333 DEGs from estradiol treatment (Table 1; Fig. 2A). The testosterone DEGs were enriched for metabolic (lipid metabolism, organic acid metabolism, bile acid biosynthesis), development, and immune response pathways (Table 1). The estradiol liver DEGs showed enrichment for metabolic (organic acid metabolism, carboxylic acid metabolism) and immune pathways (complement and coagulation).

In contrast to liver, we found that the effect of estradiol treatment was more profound (2029 DEGs) than that of testosterone (275 DEGs) in 3WA of the inguinal adipose tissue (Table 2; Fig. 2D). The estradiol DEGs were enriched for protein metabolism, focal adhesion, and transport pathways. Testosterone DEGs were enriched for cell–cell adhesion, development, regulation of transcription, and protein signaling pathways.

Overall, both estradiol and testosterone affected genes involved in metabolism, development, and immune function. However, estradiol primarily affected these processes in the adipose tissue, whereas testosterone showed influence in the liver.

### Genes and pathways affected by gonadal sex

In the liver, 3WA analyses revealed 93 DEGs influenced by gonadal sex when testosterone and blank treatment groups were considered, and 209 DEGs in the analysis of estradiol and blank groups (Table 1). These genes were enriched for immune/defense response and lipid metabolism pathways. By 2WA, we found that gonadal sex has the strongest influence on inflammatory and metabolism genes in the absence of hormones (blank group; 115 DEGs), but the effect was reduced by estradiol treatment (53 DEGs) and minimized by testosterone treatment (9 DEGs) (Table 1; Fig. 2B).

For the inguinal adipose tissue, gonadal sex had more than twice as many DEGs as in liver tissue in the 3WA analysis (cf. Fig. 2A and 2D). Further dissection of the gonadal sex effect in individual hormonal treatment groups in a 2WA analysis showed that the effects of gonadal sex were strongest in the estradiol group (400 DEGs), followed by the testosterone group (161 DEGs), and last by the blank group (70 DEGs) (Fig. 2E). Genes affected by gonadal sex are mainly relevant to developmental processes, whereas arginine and proline metabolism genes were also affected in the estradiol group and cancer-related genes affected in the testosterone group.

These results support the importance of gonadal sex in regulating development, metabolic, and immune processes in both tissues. However, in the liver, hormonal treatments minimized the effects of gonadal regulation of gene expression, whereas in the adipose tissue, hormones amplified the gonadal influence on gene expression. In both tissues, the gonadal sex effect was more prominent in the estradiol-treated group than in the testosterone-treated group (cf. Fig. 2B and 2E).

### Genes and pathways affected by sex chromosome complement

In both the 3WA and 2WA analyses, 10 or fewer genes were found to be significantly affected by sex chromosome complement at FDR < 0.05 in the liver (Table 1; Fig. 2C) and 10–22 DEGs were influenced by sex chromosomes in the adipose tissue (Table 2; Fig. 2F). These genes were mainly sex chromosome genes known to show sex differences, including *Xist*, *Ddx3y*, *Kdm6a*, *Hccs*, *Cited1*, *Tlr7*, and *Eif2s3x/y* (Chen et al. 2012; Berletch et al. 2015; Golden et al. 2019; Itoh et al. 2019). However, autosomal genes were also influenced by sex chromosome type in both liver (e.g., *Ntrk2* and *H2-DMb1*) and adipose tissue (e.g., *Pals1*, *Esrp1*, and *Dnai1*). Genes influenced by sex chromosome complement are involved in inflammation/immune response (*Tlr7*, *H2-Dmb1*, *Cited1*), GPCR signaling (*Esrp1*), metabolism (*Hccs*), and cell junction organization (*Pals1*).

### Genes and pathways affected by interactions of sex-biasing factors

The interactions among the sex-biasing factors are supported by numerous DEGs with significant effects from the interaction terms in the ANOVA analyses (FDR < 0.05) (Supplemental Table S2). For instance, in adipose tissue, 31 DEGs were affected by interactions between estradiol and gonad type. These DEGs were enriched in pathways such as VLDL particle assembly and regulation of leukocyte chemotaxis. DEGs *Dnai1* and *Cited1* were expressed in female gonads (XXF or XYF) when no sex hormones were provided; genes such as *Ctns*, *Slc2a3*, *S100a14*, and *Ier3* showed a significant increase in expression when estradiol treatment was provided to female gonads (Supplemental Fig. S3). In the liver, fewer genes showed significant interaction effects between pairs of sex-biasing factors (FDR < 0.05). For instance, expression of *Cyp3a41a*, *Sult3a1*, and *Cyp17a1* was down-regulated by testosterone in mice with female gonads; *Obp2a* expression was up-regulated by testosterone in mice with male gonads; expression of *Igfbp2* was up-regulated by testosterone on female gonads but down-regulated by testosterone on male gonads (Supplemental Fig. S4).

### Comparison of mouse DEGs affected by sex-biasing factors with human sex-biased genes

To cross-validate the DEGs identified in our FCG mouse model, we compared them with sex-biased genes identified in human GTEx studies of liver (Supplemental Table S4) and adipose tissues (Supplemental Table S5; Oliva et al. 2020). We found that 80 of 500 sex-biased genes (16%) in GTEx liver and 116 of 500 sex-biased genes (23.2%) in GTEx adipose tissue were identified as DEGs affected by one or more sex-biasing factors in our FCG model. It is important to note the key difference between studies: the sex-biased genes in GTEx are the results of the combined effects of all sex-biasing factors, whereas our FCG mouse study focuses on the effect of individual sex-biasing factors.

Because GTEx studies cannot isolate specific sex-biasing factors, our FCG model suggests the particular factors contributing to the sex-biased genes found in humans. For instance, in adipose tissue, the GTEx female-biased genes *ASAH1*, *PRDX2*, and *LOXL1* might be explained by an effect of estradiol. In contrast, the male-biased adipose gene *HSD11B1* in GTEx can be explained in the FCG by the effect of testosterone (Supplemental Fig. S5). In the liver, the human male-biased genes *ADH4*, *GNA12*, and *HSD17B12* can be explained in our mouse model by an effect of testosterone, whereas the female-biased human genes *AS3MT*, *ZFX*, and C*XCL16* were found to be affected by estradiol in FCG mice (Supplemental Fig. S6). Therefore, the FCG mice not only can recapitulate certain sex-biased genes in human studies but suggest the specific sex-biasing factors that contribute to the sex bias.

### Coexpression modules affected by each sex-biasing factor

The preceding DEG analyses focused on genes that were individually influenced by sex-biasing factors as well as their interactions. Sets of genes that are highly coregulated or coexpressed can offer complementary information on coordinated gene regulation by sex-biasing factors that might be missed by the DEG-based analyses. To this end, we constructed gene coexpression networks for each tissue using MEGENA (Methods) and identified 326 liver and 131 adipose coexpression modules. The first PCs of the coexpression modules were assessed for influence by sex chromosome, gonadal sex, and hormonal treatment factors using three-, two-, and one-way ANOVAs (Fig. 2G,H). We confirmed the large effect of hormonal treatment in regulating modules enriched for diverse biological pathways. In the liver, testosterone affected modules involved in metabolism (RNA, lipid, protein), development, protein assembly, chemical response, immune system (inflammation, adaptive immune response), apoptosis, and transcription/translation. In adipose tissue, estradiol influenced modules related to focal adhesion, development, metabolism (protein, lipid, oxidative phosphorylation), immune system (complement and coagulation), and translation.

Gonadal sex also showed considerable influence on liver modules related to protein metabolism/assembly, development, stress/immune response, apoptosis, and transcription/translation regulation, whereas in adipose tissue gonadal sex mainly affected developmental and focal adhesion processes, and to a lesser degree, lipid metabolism, biological oxidation, and intracellular signaling modules (Fig. 2G).

The coexpression network analysis also confirmed the limited effect of sex chromosomal variation on altering coexpression modules (Fig. 2G,H). However, in adipose tissue, sex chromosomes showed weak effects on modules related to lipid metabolism and intracellular signaling when the estradiol and blank groups were considered, but not when the testosterone group was included (Fig. 2H).

Overall, the gene coexpression network analysis offered clearer patterns of tissue specificity and functional specificity of each sex-biasing factor compared to the DEG-based analysis.

### Bulk tissue deconvolution to understand cellular composition changes through sex-biasing factors

To explore whether the DEGs and pathways/modules identified in FCG can be explained by cellular composition changes affected by each sex-biasing factor, we performed cell composition deconvolution analysis on the bulk tissue transcriptome data using CIBERSORTx based on single-cell reference data sets of the corresponding tissues (Methods). We subsequently assessed the hormonal, gonadal, and sex chromosomal effects on individual cell types.

In both the liver (Supplemental Fig. S7) and adipose tissue (Supplemental Fig. S8), hormones affected the largest number of cell types in terms of their abundance, including various immune cell populations such as the hepatocellular stellate cells (HSCs) and neutrophils in the liver, and macrophages, CD4 T cells, dendritic cells, and antigen presenting cells in adipose tissue. Hormones also affected dividing cell populations and endothelial cells in both tissues. These cell populations affected by hormones support the DEGs and pathways involved in immune functions and development that are influenced by the same sex-biasing factor. Similar to the findings based on DEG and pathways analysis, the gonadal effect on cell populations is also dependent on the tissue and other sex-biasing factors: female gonads showed increases in hepatocytes, endothelial, and HSCs in the liver on an XX background, whereas male gonads showed an increase in macrophage proportion in adipose tissue on an XY and testosterone background. Last, the sex chromosome effect can be noted in immune cell populations, but it is generally dependent on the interactions with other sex factors. Overall, the changes in cellular composition support the changes in the pathways highlighted through our DEGs and coexpression modules including immune, developmental, and metabolic signals in both tissues.

### Effect of hormonal treatment on gene expression direction across genotypes

Because of the dominant effect of hormonal treatment compared to gonadal sex or sex chromosome differences based on the preceding analyses, we further investigated the differences between testosterone and estradiol treatments in terms of the gene sets they target and the direction of gene expression change within and between tissues.

**Figure 3.**
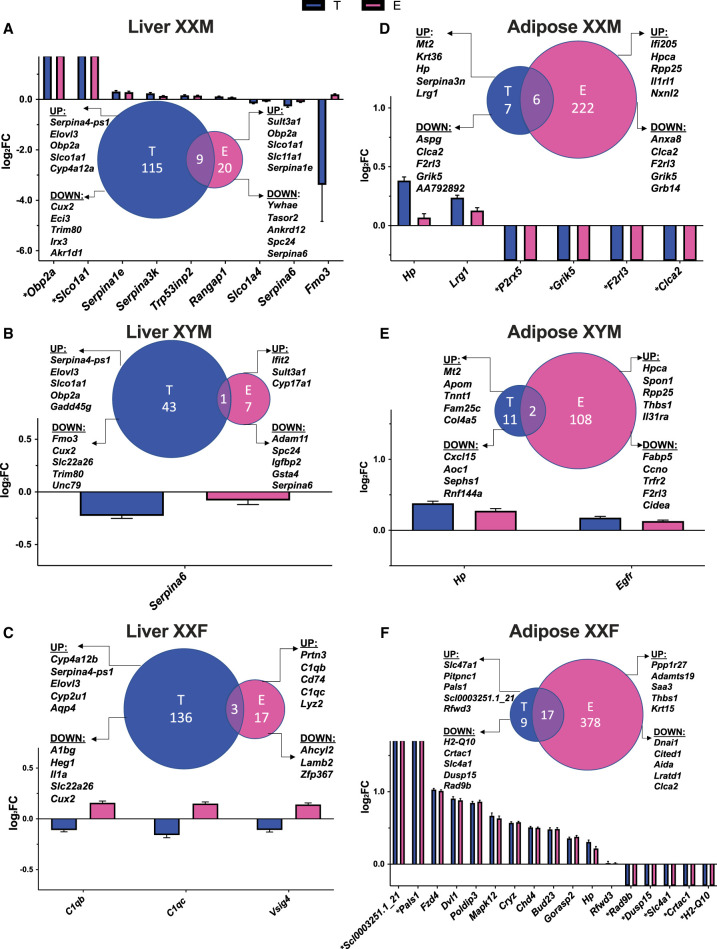
Venn diagrams of DEG comparisons and bar graphs of overlapping DEGs between estradiol (E vs. blank, abbreviated as E) and testosterone (T vs. blank, abbreviated as T) treatment for each genotype in liver and adipose. (*A*) Liver XXM. (*B*) Liver XYM. (*C*) Liver XXF. (*D*) Adipose XXM. (*E*) Adipose XYM. (*F*) Adipose XXF. The bar graphs focused on the DEGs that passed an FDR < 0.05 and were overlapping between testosterone and estradiol treatment for each genotype and tissue. To understand the effects of each hormone, we plotted the log_2_ fold change (log_2_FC) of the hormonal effects. The Venn diagrams showcase comparison of DEGs of T effect versus E effect, as well as the top five up- and down-regulated genes for T or E in liver or adipose tissue for each genotype. There was no statistically significant overlap between any comparisons in the Venn diagrams. (*) Genes that are not expressed in one of the comparison groups and thus have infinite log_2_FC values.

Comparing groups of DEGs regulated by testosterone or estradiol in the 3WA (Supplemental Fig. S9), 226 overlapped in the liver and 383 overlapped for adipose tissue. However, estradiol DEGs in individual genotypes had limited overlap with those caused by testosterone in 1WA (Fig. 3). In particular, for the XYF mouse, we found no overlapping DEGs in either the liver or adipose DEGs between testosterone and estradiol (Supplemental Fig. S10). For other genotypes, the overlapping DEGs in the liver (Fig. 3A–C) and adipose tissues (Fig. 3D–F) mostly had consistent directions of expression changes between hormones, except that flavin containing monooxygenase 3 (*Fmo3*; important for the breakdown of nitrogen-containing compound) in XXM liver (Fig. 3A) and all the shared DEGs in XXF liver (*C1qb*, *C1qc*, and *Vsig4*; complement pathway genes) (Fig. 3C) were affected by testosterone (down) and estradiol (up) oppositely.

### Identification of potential regulators of sex-biasing factors

#### Transcription factor network analysis

**Figure 4.**
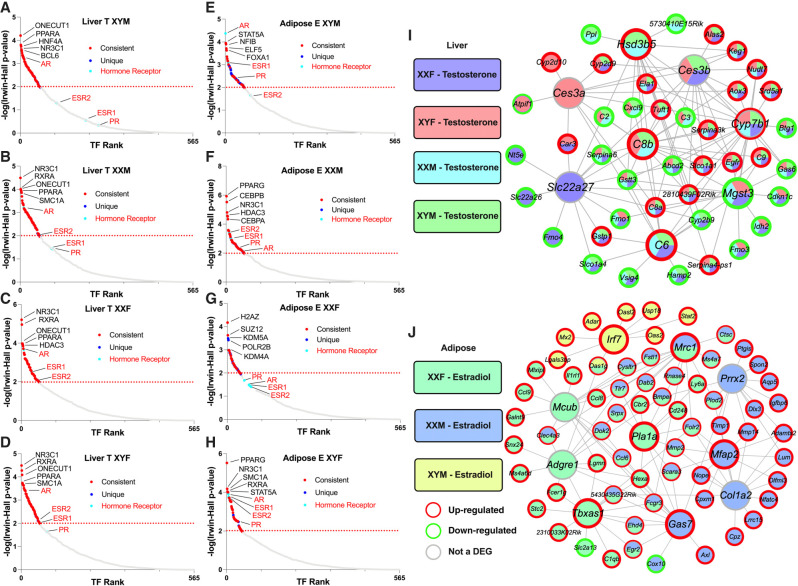
Transcription factor analysis (*A*–*H*) and key driver analysis (*I*,*J*) of DEGs informed by estradiol and testosterone treatment in liver and adipose. (*A*–*D*) TF analysis for liver. (*E*–*H*) TF analysis for adipose. For the TF network, we used DEGs (FDR < 0.05) from our 1WA for testosterone and estradiol treatment analysis using the BART tool, in which a TF was considered significant by an Irwin–Hall *P* < 0.01 analogous to −log_10_(*P*-value) = 2. Red dots signify the TF is present in at least one other genotype and the blue dots signify if the TF is only present in the given genotype. Turquoise dots and red font denote a hormonal receptor relevant to testosterone and estradiol. Labeled TFs showcase the top five by rank and additional hormonal receptors. (*I*) Liver gene regulatory network (GRN). (*J*) Adipose GRN. For GRN construction, we overlaid DEGs (FDR < 0.05) from our post hoc 1WA for testosterone and estradiol treatment onto our previously built adipose and liver Bayesian networks utilizing a KDA analysis from the Mergeomics package. We visualized the top five KDs for the testosterone or estradiol DEGs from each genotype group. KDs are labeled as larger nodes and DEGs as smaller nodes. Direction of DEGs is annotated with red or green borders for up-regulation or down-regulation, respectively.

To understand the regulatory cascades that explain the large numbers of sex-biased genes affected by hormone treatments (Fig. 3), we performed transcription factor (TF) analysis using as input DEGs that passed an FDR < 0.05 from 1WA specific to testosterone effects in the liver and estradiol effects in adipose tissue (Tables 1, 2). For the testosterone liver DEGs, we identified 67, 66, 60, and 62 TFs for XYM, XXM, XXF, and XYF, respectively (Fig. 4A–D; Supplemental Table S6). As expected, we captured gonadal hormone receptors including androgen receptor (AR) as a highly ranked TF in all genotypes and estrogen receptors (ESR1, ESR2, ESRRA) to be TFs with lower rank. We also found nuclear receptor subfamily 3 (NR3C1; the glucocorticoid receptor important for inflammatory responses and cellular proliferation) to be among the top five TFs for all four genotypes and the top-ranked TF for XXF and XYF, which is consistent with a female bias for this TF found in the GTEx study (Oliva et al. 2020). A number of circadian rhythm TFs were found throughout all genotypes in the liver including CRY1, CRY2, PER1, and PER2, which is consistent with sex differences in body clocks (Anderson and FitzGerald 2020). Additional consistent TFs for testosterone effect in liver across multiple genotypes, where sex bias has been documented previously, include FOXA1/2, XBP1, HNF4A, SPI1, and CTCF.

An analysis of TFs that may mediate estradiol effects in adipose tissue identified 64, 61, 44, and 53 TFs for XYM, XXM, XXF, and XYF, respectively (Fig. 4E–H; Supplemental Table S7). We found ESR1 and ESR2 as consistent TFs throughout the genotypes, except for XYF, where no classical estradiol or androgen receptor TF was captured. We also identified AR as a top TF in XYM and XYF. Notably, we found many TFs across our genotypes to be consistent with the TFs for female-biased genes in the Anderson et al. (2020) human adipose study. Of their top 20 ranked TFs for female-biased genes, we found 17 in our results for estradiol treatment in our genotypes, including ESR1, H2AZ, SUZ12, KDM2B, CEBPB, and PPARG. The top TFs were generally consistent across genotypes, except KDM5A, POLR2B, KMT2C, and CLOCK were particular to XXF.

When looking into the TFs that mediate estradiol's effects in XYM for potential male-biased regulation in adipose tissue, we found matches with 13 of the top 20 TFs from the Anderson et al. (2020) human adipose study. These included AR, CTCF, SMC1A, EZH2, ESR1, RAD21, and TP63, and many were also consistent in additional mouse (Anderson et al. 2020; Matthews and Waxman 2020) and human studies including GTEx (Anderson et al. 2020; Oliva et al. 2020).

#### Gene regulatory network analysis

An alternative and complementary approach to the preceding TF analysis is to use a gene regulatory network approach to decipher the key drivers (KDs) that may drive sex-biased gene alterations in each genotype based on the DEGs found in 1WA (Tables 1, 2). These KDs did not overlap with the TFs identified above owing to the incorporation of genetic regulatory information in network construction.

In the liver (Fig. 4I), we saw overlapping KDs for testosterone DEGs across all four genotypes. *Cyp7b1*, which is important in converting cholesterol to bile acids and metabolism of steroid hormones, was among the top five KDs for all genotypes. *Mgst3* (involved in inflammation), *C6* and *C8b* (complement genes), and *Ces3b* (xenobiotics detoxification) were top five KDs for three of the four genotypes (Fig. 4I). We also identified KDs specific to particular genotypes (Supplemental Table S8) such as *Ces3a* (xenobiotics detoxification) for female gonads, *Slc22a27* (anion transport) for XXF, *Serpina6* (inflammation) for XYF, and *Hsd3b5* (steroid metabolism) for male gonads. Among these KDs, *Slc22a27* was previously found to be expressed predominantly in females, and *Hsd3b5* and *Cyp7b1* were male specific (Adams et al. 2015), thus agreeing with our results.

For estradiol, 31 KDs were found for adipose tissue DEGs from the XXF, XXM, and XYM genotypes (Fig. 4J; Supplemental Table S9). The KDs included *Mrc1* (response to infection), which is the only overlapping top KD between genotypes XXF and XXM. KDs that were more highly ranked for XXM but still statistically significant in XXF included genes involved in extracellular matrix organization (*Prrx2*, *Mfap2*, *Col1a2*, and *Gas7*), and those specific to XXF are relevant to lipid synthesis/metabolism (*Tbxas1*, *Pla1a*) and immune function (*Adgre1* and *Mcub*). *Irf7* is the only KD for XYM, which has been recently suggested to be a TF in adipocytes with roles in adipose tissue immunity as well as obesity (Kuroda et al. 2020).

### Disease association of the genes affected by sex-biasing factors

**Figure 5.**
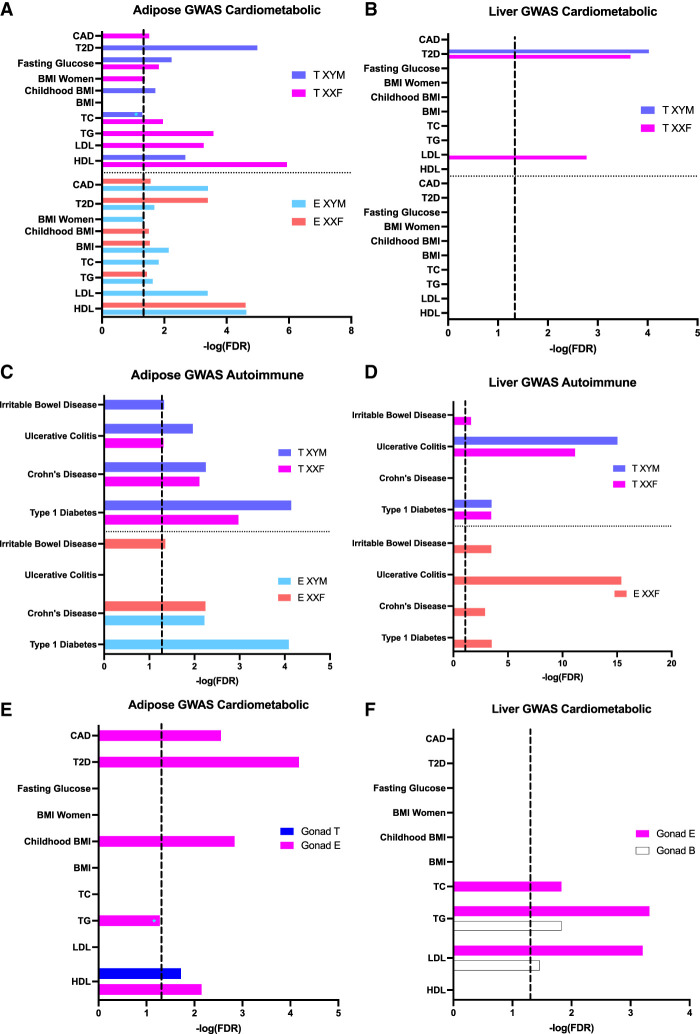
Bar graphs showing enrichment of the hormone DEGs (*A*–*D*) and gonadal DEGs (*E*,*F*) for known cardiometabolic and autoimmune diseases based on MSEA analysis. The cardiometabolic category included coronary artery disease (CAD), type 2 diabetes (T2D), fasting glucose level, BMI in women, BMI during childhood, BMI, total cholesterol (TC), triglyceride (TG), low-density lipoprotein (LDL) cholesterol, and high-density lipoprotein (HDL) cholesterol (*A*,*B*). The autoimmune category included irritable bowel disease (IBD), ulcerative colitis (UC), Crohn's disease (CD), and type 1 diabetes (T1D) (*C*,*D*). (*A*) Association of adipose testosterone (T) and estradiol (E) DEGs with cardiometabolic diseases/traits. (*B*) Association of liver T and E DEGs with cardiometabolic diseases/traits. (*C*) Association of adipose T and E DEGs with autoimmune diseases. (*D*) Association of liver T and E DEGs with autoimmune diseases. (*E*) Association of adipose gonadal DEGs with cardiometabolic diseases/traits. (*F*) Association of liver gonadal DEGs for cardiometabolic diseases/traits. (*A*–*D*) Hormone DEGs at an FDR < 0.05 derived from the post hoc one-way ANOVA were tested against genetic association signals with cardiometabolic and autoimmune diseases and traits. (*E*,*F*) Gonadal DEGs at an FDR < 0.05 from two-way ANOVA were tested against genetic association signals with cardiometabolic diseases. Dotted line signifies FDR < 0.05; (*) enrichment minimally below the FDR < 0.05 cutoff.

Finally, to test the disease relevance of the genes affected by sex-biasing factors, we used a marker set enrichment analysis (MSEA; details in Methods) to detect whether the DEGs highlighted in the 1WA overlap with genes previously identified to have SNPs associated with human diseases/pathogenic traits by GWAS. In brief, we mapped each of the GWAS SNPs to genes using liver and adipose eQTLs to represent disease-associated genes informed by GWAS. The mouse orthologs of these human GWAS disease genes were then compared with sex-biased DEGs from FCG to connect the genes affected by individual sex factors with human disease genes. Of the 73 disease/traits screened for which full GWAS summary statistics were available, we focused on two broad categories: “cardiometabolic” (Fig. 5A,B) and “autoimmune” (Fig. 5C,D), both of which are known to show sex differences. For hormone DEGs, we focused on those that are directly relevant to the general human population to understand how testosterone or estradiol can affect disease outcomes on XYM (physiological males) or XXF (physiological females).

#### Disease association for hormone DEGs

When cardiometabolic diseases were considered, testosterone and estradiol DEGs in the adipose tissue from both the XYM and XXF genotypes showed extensive disease associations (Fig. 5A; Supplemental Table S10). In contrast, liver DEGs for both hormones showed limited cardiometabolic associations, with specificity of testosterone DEGs for both T2D and LDL but no association for estradiol DEGs (Fig. 5B; Supplemental Table S11). In terms of autoimmune diseases, testosterone DEGs in both the adipose (Fig. 5C; Supplemental Table S12) and liver (Fig. 5D; Supplemental Table S13) from both XXF and XYM genotypes showed enrichment for disease associations. The estradiol DEGs in both tissues, however, had a genotype-dependent pattern for disease association. In particular, estradiol liver DEGs from XYM had no association with autoimmune diseases but DEGs in XXF were associated with all autoimmune diseases.

Overall, adipose DEG sets altered by both hormones showed enrichment for both cardiometabolic and autoimmune processes. For liver hormone DEGs, the most significant associations were with autoimmune diseases, whereas T2D and LDL associations were also identified for liver testosterone DEGs.

#### Disease association for gonadal sex DEGs

We also used MSEA to detect whether gonadal DEGs highlighted in 2WA (FDR < 0.05) overlap with human disease genes informed by GWAS. For both adipose tissue (Fig. 5E; Supplemental Table S14) and liver (Fig. 5F; Supplemental Table S15), the gonadal DEGs on an estradiol background showed associations with cardiometabolic diseases or traits, whereas gonadal DEGs on a testosterone or blank background had limited or no disease association.

#### Disease association for sex chromosome DEGs and interaction DEGs

Because of the low number of DEGs captured for the sex chromosome effect or interactions among the sex-biasing factors, no enrichment results are possible through MSEA; therefore, we queried whether these DEGs have been previously implicated in human diseases by overlapping the DEGs at FDR < 5% with candidate genes from the GWAS catalog for 2203 traits. Both adipose tissue and liver DEGs demonstrating sex chromosome effects, or interactions between gonad and hormone, or interactions between sex chromosome and gonad, overlapped with GWAS candidates for numerous cardiometabolic and autoimmune diseases (Supplemental Tables S16–S18).

## Discussion

The variation in physiology and pathophysiology between sexes is established via the modulatory effects of three main classes of sex-biasing agents. The manifestations of these sex-dependent modulators impact disease incidence and severity, including metabolism-related diseases and autoimmune diseases (Voskuhl and Gold 2012; Link and Reue 2017). In this study, we separated the effects of these sex-biasing components using the FCG model, thus enabling the analysis of each contributing factor as well as their interactions in altering gene expression in inguinal adipose and liver tissues, which are relevant in systems metabolism and immunity.

**Figure 6.**
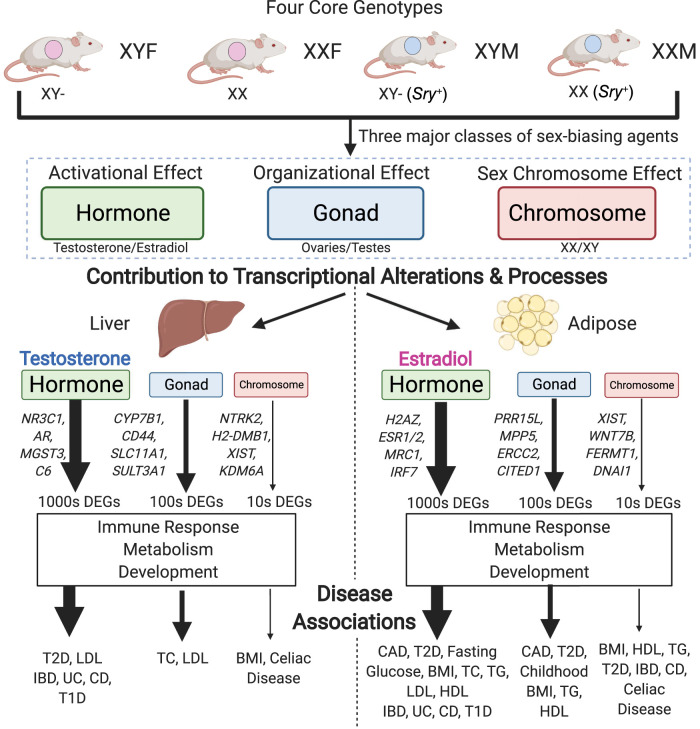
Study summary. Using the FCG model, we separated the effects of three major classes of sex-biasing agents and uncovered their relative contribution to transcriptional alterations in the liver and adipose tissue, the resulting biological processes enriched, and finally the diseases associated.

Our data revealed distinct patterns between tissues in the relative contribution of each sex-biasing factor to gene regulation (Tables 1, 2; Fig. 6). In particular, the liver transcriptome is mainly affected by acute effects of testosterone, followed by acute effects of estradiol, organizational effect of gonadal sex, and sex chromosome complement, whereas inguinal adipose gene expression is primarily regulated by acute effects of estradiol, followed by gonadal sex, acute effects of testosterone, and sex chromosome complement. The genes and pathways regulated by the sex-biasing factors are largely different between factors, although metabolic, developmental, and immune functions can be regulated by both activational effects of sex hormones and gonadal sex (organizational effects). Sex chromosome effects were primarily associated with genes that reside on X and Y Chromosomes, along with a handful of autosomal genes involved in inflammation and metabolic processes that are downstream from the sex-biasing effects of X and Y genes. Cell deconvolution analysis supports that sex-biasing factors influence the proportion of diverse cell populations such as immune cells, hepatocytes, and dividing cells, suggesting that cellular composition changes may partially explain the observed genes and pathways. Last, the liver and adipose tissue genes affected by the sex-biasing factors were found to be downstream targets of numerous TFs and network regulators, not just the sex hormone receptors, and show association with human cardiometabolic and autoimmune diseases.

Previously, sex differences in the liver transcriptome have been largely attributed to sex differences in the circadian rhythm and levels of growth hormone, which are established because of perinatal organizational masculinization of hypothalamo-pituitary mechanisms controlling growth hormone (Mode and Gustafsson 2006; Waxman and Holloway 2009; Sugathan and Waxman 2013). Genes regulated in this manner would be expected to appear in the gonadal-effect DEGs. Our results suggest, however, that the acute activational effects of gonadal hormones might be a more important influence, because of the larger number of testosterone or estradiol DEGs compared to gonad DEGs. Our results are in line with previous evidence that removal of gonadal hormones in adulthood eliminates most sex differences in mouse liver gene expression (van Nas et al. 2009; Norheim et al. 2019), and that liver-specific knockout of estrogen receptor alpha or androgen receptor altered genes that underlie sex differences in the liver transcriptome (Zheng et al. 2018). It is possible that the effects of gonadal steroids during adulthood are required for some of the organizational effects of testosterone mediated via growth hormone action. In contrast to liver, gonadectomy does not eliminate sex differences in the adipose transcriptome (Norheim et al. 2019), which agrees with our finding that the organizational effects of gonads play a strong role, in addition to estradiol, in adipose gene regulation. The striking tissue specificity for each of the sex-biasing factors observed here highlights that individual tissues have unique sex-biased regulatory mechanisms.

We found that the gonadal sex factor primarily affects developmental pathways, cell adhesion, and metabolic pathways in adipose (Table 2; Fig. 2H), which corroborates past evidence indicating that early gonadal sex status and associated hormonal release play critical roles in the development of sex differences and disease outcomes (Leung et al. 2004; Varlamov et al. 2012; Shen and Shi 2015).

Compared to the organizational gonadal sex effects and activational hormone effects, the sex chromosome effects were minimal, and no coherent pathways were found for the sex chromosome–driving DEGs (Tables 1, 2) or coexpression modules (Fig. 2G,H). The DEGs include those known to escape X inactivation (*Kdm6a*, *Eif2s3x*, *Ddx3x*) (Chen et al. 2012; Berletch et al. 2015) and their Y paralogs (*Eif2s3y*, *Ddx3y*). The X escapees are expressed higher in XX than XY cells, causing sex differences in several mouse models of metabolic, immune, and neurological diseases (Kaneko and Li 2018; Itoh et al. 2019; Davis et al. 2020; Link et al. 2020).

As our comparative analysis of the three classes of sex-biasing factors clearly determined that the activational effects of gonadal hormones are the dominant factors, we further investigated potential upstream regulatory factors that may control the sex-biased genes, using a gene regulatory network analysis and a TF analysis, revealing both expected and novel findings. In concordance with the importance of hormonal effects and consistent with recent human studies including GTEx searching for tissue-specific sex bias (Anderson et al. 2020; Oliva et al. 2020), TFs for sex hormone receptors (AR and ESR1/2) were captured in the majority of genotypes (Fig. 4A–H). Beyond the major hormonal receptors, within the liver numerous circadian-related TFs were captured (PER1, PER2, CRY1, and CRY2). Although it is known that males and females have differing biological clocks (Anderson and FitzGerald 2020), the contribution of hormones particularly in this rhythm is far from fully elucidated, and our findings support that hormones need to be taken into account in liver circadian rhythm studies. In adipose tissue for estradiol treatment, however, we found that the XXF genotype has no significant signal for ERs, which may imply that estradiol's major contribution in adipose gene regulation is more importantly through TFs such as H2AZ, which has been shown to be essential for estrogen signaling and downstream gene expression (Gévry et al. 2009). In addition to TFs, we used a GRN analysis, revealing non-TF regulators. For the liver GRN (Fig. 4I), key driver genes for testosterone DEGs are involved in immune processes (*Mgst3*, *C6*, *C8b*), steroid metabolism (*Cyp7b1* and *Hsd3b5*), and xenobiotic detoxification (*Ces3b* and *Ces3a*). In adipose tissue (Fig. 4J), there were far fewer shared key drivers for estradiol DEGs across genotypes relative to the results in the liver with testosterone treatment, indicating that estradiol has more finely tuned interactions with the gonadal sex and sex chromosome genotypes than the broad effect of testosterone.

Last, to provide context to the health relevance of the liver and adipose sex-biasing DEG sets, we looked for GWAS association of these genes with human diseases/traits. We found that hormone-affected genes in adipose tissue were enriched for genetic variants associated with numerous cardiometabolic diseases/traits, but the enrichment was weaker for the liver DEGs (Fig. 5A,B). Another important area of sex difference is found within autoimmunity, which occurs more in females (Mauvais-Jarvis et al. 2020). Although both adipose and liver DEGs from multiple hormone-genotype combinations were enriched for autoimmune diseases, the liver DEGs, particularly those from the XXF genotype, had more prominent autoimmune association. Beyond the hormonal DEG enrichment in human disease/trait, we also found that DEGs caused by gonad type from both adipose and liver are involved in cardiometabolic disease (Fig. 5E,F). Finally, despite minimal DEGs captured for the sex chromosome effect as well as the interactions between sex-biasing factors, we found overlap of these DEGs with various disease traits. The DEGs underlying disease associations may explain the differential susceptibility of males and females to these major diseases and warrant further investigation to distinguish risk versus protection through the genes identified in this study.

The analyses presented in this study show an extensive dissection of the relative contribution of three classes of sex-biasing factors on liver and adipose gene expression, their associated biological processes and regulators, and their potential contribution to disease. Importantly, many of the genes identified in our study were replicated in independent human studies such as GTEx, and our mouse study offers unique insights into the particular sex-biasing factors (hormones, sex chromosomes, or gonads) that likely contribute to the sex-biased gene expression in humans. Despite retrieving numerous new insights, we acknowledge the following limitations. First, gonadectomy and subsequent treatment of hormones may have caused activational effects that do not match the effects of endogenous physiological changes in the same hormones, leading to more predominant activational effects being observed. Second, the relative effects of testosterone and estradiol are affected by the doses of each hormone used. Testing additional doses is required for detailed comparison of effects of the two hormones. Third, we used DEG counts as a measure of overall effect size to compare the various sex-biasing factors, which may be influenced by sample size and statistical power. Therefore, caution is needed when interpreting the results. However, the sample sizes are comparable across sex-biasing factors and are adequate for mouse transcriptome studies with sufficient statistical power (Pawitan et al. 2005; Lin et al. 2010). Fourth, the comparison of mice with testes versus ovaries does not map perfectly onto mice that had organizational effects of testicular versus ovarian secretions because of the potential effects of the *Sry* transgene, which was present in tissues only of mice with testes. Last, only liver and inguinal adipose tissues were investigated, and other tissues warrant examination in future studies.

Overall, our data revealed tissue-specific differential gene expression resulting from the three sex-biasing factors, thereby distinguishing their relative contributions to the differential expression of key genes in a variety of clinically significant pathways including metabolism, immune activity, and development. Importantly, in addition to establishing the critical influence of hormones and their effect on the transcriptome in a tissue-specific manner, we also uncovered and highlighted the underappreciated role of the sex chromosomal effect and organizational gonadal effect as well as interactions among sex-biasing factors in global gene regulation. Our findings offer a comprehensive understanding of the origins of sex differences, and each of their potential associations with health and disease.

## Methods

### Animals

Mouse studies were performed under approval of the UCLA Institutional Animal Care and Use Committee. We used FCG mice on a C57BL/6J B6 background (B6.Cg-TgSry2Ei Srydl1Rlb/ArnoJ, Jackson Laboratories stock 10905; backcross generation greater than 20), bred at UCLA (De Vries et al. 2002; Burgoyne and Arnold 2016). Gonadal females and males were housed in separate cages and maintained at 23°C with a 12:12 light:dark cycle.

A total of 60 FCG mice, representing four genotypes (XXM, XXF, XYM, XYF), were gonadectomized (GDX) at 75 d of age and implanted immediately with medical-grade Silastic capsules containing Silastic adhesive only (blank control [B], testosterone [T], or estradiol [E]) (for details, see Supplemental Methods). Mice were euthanized 3 wk later; liver and inguinal adipose tissues were dissected, snap frozen in liquid nitrogen, and stored at −80°C for RNA extraction and Illumina microarray analysis.

### RNA isolation, microarray hybridization, and quality control

RNA from liver and inguinal adipose tissue was isolated using TRIzol (Invitrogen). Individual samples were hybridized to Illumina MouseRef-8 Expression BeadChips (Illumina) by Southern California Genotyping Consortium (SCGC) at UCLA. Two adipose samples were removed from the total of 60 after RNA quality test (degradation detected). Principal component analysis (PCA) was used to identify three outliers among the adipose sample, which were removed from subsequent analyses. PCA was conducted using the prcomp R package (R Core Team 2020) with the correlation matrix (for details, see Supplemental Methods).

### Identification of differentially expressed genes affected by individual sex-biasing factors

To identify differentially expressed genes (DEGs), we conducted three-way ANOVA (3WA), two-way ANOVA (2WA), and one-way ANOVA (1WA) using the aov R function. The 3WA tested the general effects of three factors of sex chromosomes, gonad, and hormonal treatments, as well as their interactions. The 2WA tested the effects of sex chromosomes and gonads as well as their interaction within each hormonal treatment group (T, E, or B) separately. For 1WA, we tested the effects of T (comparing T vs. B) and E (comparing E vs. B) within each genotype. Multiple testing was corrected using the Benjamini–Hochberg (BH) method, and significance level was set to FDR < 0.05 to define significant DEGs (for details, see Supplemental Methods).

### Coexpression network construction and identification of differential modules affected by individual sex-biasing factors

We used the multiscale embedded gene coexpression network analysis (MEGENA) (Song and Zhang 2015), a method similar to WGCNA (Langfelder and Horvath 2008), to recognize modules of coexpressed genes affected by the three different sex-biasing factors (for details, see Supplemental Methods). The influence of each sex-biasing factor on the resulting modules was assessed using the first principal component of each module to represent the expression of that module, followed by 3WA, 2WA, 1WA tests, and FDR calculation as described under the DEG analysis section to identify differential modules (DMs) at FDR < 0.05 that are influenced by each sex-biasing factor.

### Annotation of the pathways overrepresented in the DEGs and DMs

For each of the DEG sets and DMs that were significantly affected by any of the sex-biasing factors, we conducted pathway enrichment analysis against Gene Ontology (GO) Biological Processes and KEGG pathways derived from MSigDB using Fisher's exact test, followed by BH FDR estimation (for details, see Supplemental Methods).

### Gene regulatory network analysis

To predict potential regulators of the sex-biased DEGs, we used the key driver analysis (KDA) function of the Mergeomics pipeline (Shu et al. 2016) and liver and adipose Bayesian networks. In brief, the Bayesian networks were built from multiple large human and mouse transcriptome and genome data sets (Yang et al. 2006; Wang et al. 2007; Emilsson et al. 2008; Schadt et al. 2008; Tu et al. 2012). To identify the key driver (KD) genes within these networks, the KDA uses a χ^2^-like statistic to identify genes that are connected to a significantly larger number of DEGs than what would be expected by random chance (for details, see Supplemental Methods). KDs were considered significant at FDR < 0.05, and the top KD subnetworks were visualized using Cytoscape (Shannon et al. 2003).

### Transcription factor analysis

To predict transcription factors (TFs) that may regulate the sex-biased DEGs sets, we used the binding analysis for regulation of transcription (BART) computational method (Wang et al. 2018). We followed the tool's recommendation of a minimum of 100 DEGs as input and an Irwin–Hall *P*-value cutoff (*P* < 0.01) to identify TFs.

### Marker set enrichment analysis to connect sex-biasing DEGs with human diseases or traits

To assess the potential role of the DEGs affected by each of the sex-biasing factors in human diseases, we collected the summary statistics of human GWAS for 73 diseases or traits that are publicly available via GWAS catalog (MacArthur et al. 2017). SNPs that have linkage disequilibrium of r^2^> 0.5 were filtered to remove redundancies. To map GWAS SNPs to genes, we used GTEx Version 7 eQTL data for liver and adipose tissues (The GTEx Consortium 2013) to derive tissue-specific genes potentially regulated by the SNPs. We then used the marker set enrichment analysis (MSEA) function embedded in Mergeomics (Shu et al. 2016) to compare the disease association *P*-values of the SNPs representing the DEGs with those of the SNPs mapped to random genes to assess whether the DEGs contain SNPs that show stronger disease associations than random genes using a χ^2^-like statistic (for details, see Supplemental Methods).

### Deconvolution of bulk liver and inguinal adipose tissue

As our reference data sets, we downloaded single-cell RNA-seq data for mouse liver (GSE166178) and mouse inguinal adipose (GSE133486) from the NCBI Gene Expression Omnibus (GEO; https://www.ncbi.nlm.nih.gov/geo/), and used CIBERSORTx (Newman et al. 2019) to impute cell fractions in each sample. Cell proportion estimates were compared across groups to identify cell types influenced by sex hormones using 1WA with post hoc analysis and by gonads or sex chromosomes using *t*-test.

## Data access

All raw and processed microarray data generated in this study have been submitted to the NCBI Gene Expression Omnibus (GEO; https://www.ncbi.nlm.nih.gov/geo/) under accession number GSE176033. R code used in the analysis is accessible via GitHub (https://github.com/XiaYangLabOrg/FCG) and as Supplemental Code.

## Supplementary Material

Supplemental Material
