# Supplementary material for: Relative contributions of sex hormones, sex chromosomes, and gonads to sex differences in tissue gene regulation
Source: Genome Res. 2022 May;32(5):807–24. doi: 10.1101/gr.275965.121 (PMC9104702; doi:10.1101/gr.275965.121)
Supplement: Supplemental Material [file supp_32_5_807__DC1.html]

Relative contributions of sex hormones, sex chromosomes, and gonads to sex differences in tissue gene regulation — Supplemental Material 

# Relative contributions of sex hormones, sex chromosomes, and gonads to sex differences in tissue gene regulation

## Supplemental Material

- Supplemental\_Table\_S1.xlsx
- Supplemental\_Table\_S2.xlsx
- Supplemental\_Table\_S3.xlsx
- Supplemental\_Table\_S4.xlsx
- Supplemental\_Table\_S5.xlsx
- Supplemental\_Table\_S6.xlsx
- Supplemental\_Table\_S7.xlsx
- Supplemental\_Table\_S8.xlsx
- Supplemental\_Table\_S9.xlsx
- Supplemental\_Table\_S10.xlsx
- Supplemental\_Table\_S11.xlsx
- Supplemental\_Table\_S12.xlsx
- Supplemental\_Table\_S13.xlsx
- Supplemental\_Table\_S14.xlsx
- Supplemental\_Table\_S15.xlsx
- Supplemental\_Table\_S16.xlsx
- Supplemental\_Table\_S17.xlsx
- Supplemental\_Table\_S18.xlsx
- Supplemental\_Code.zip
- Supplemental\_Material.docx
